# A Series of Novel Alleles of *Ehd2* Modulating Heading and Salt Tolerance in Rice

**DOI:** 10.3390/plants14020297

**Published:** 2025-01-20

**Authors:** Peng Xu, Shulei Hao, Xiaoxia Wen, Guifang Ma, Qinqin Yang, Ling Liu, Galal Bakr Anis, Yingxin Zhang, Lianping Sun, Xihong Shen, Qunen Liu, Daibo Chen, Yongbo Hong, Yuyu Chen, Xiaodeng Zhan, Shihua Cheng, Liyong Cao, Weixun Wu

**Affiliations:** 1 State Key Laboratory of Rice Biology and Breeding, China National Center for Rice Improvement, China National Rice Research Institute, Hangzhou 311400, China; 2 Key Laboratory of Northern Japonica Rice Research in Heilongjiang Province, Baoqing Northern Rice Research Center, Northern Rice Research Center of China National Rice Research Institute, Shuangyashan 155600, China; 3 Institute of Future Agriculture, Northwest Agriculture & Forestry University, Yangling 712100, China; 4 Rice Research and Training Center, Field Crops Research Institute, Agriculture Research Center, Kafrelsheikh 33717, Egypt; 5 Department of Resources and Environment, Moutai Institute, Renhuai 564507, China

**Keywords:** rice, alternative splicing, heading date, salt stress

## Abstract

Rice (*Oryza sativa* L.) is a staple crop for nearly half of the global population and one of China’s most extensively cultivated cereals. Heading date, a critical agronomic trait, determines the regional and seasonal adaptability of rice varieties. In this study, a series of mutants (*elh5* to *elh12*) exhibiting extremely late heading under both long-day (LD) and short-day (SD) conditions were identified from an ethyl methanesulfonate (EMS) mutant library. Using MutMap and map-based cloning, the causative gene was identified as a novel allele of *Ehd2*/*OsID1*/*RID1*/*Ghd10*. Functional validation through CRISPR/Cas9 knockout and complementation assays confirmed its role in regulating heading. The *elh6* mutation was found to cause intron retention due to alternative splicing. *Ehd2* encodes a Cys-2/His-2-type zinc finger transcription factor with an IDD domain and transcriptional activity in yeast. Its expression peaks in developing leaves before heading and spikes during reproductive conversion. In *elh6* mutants, delayed heading resulted from downregulating the *Ehd1*-*Hd3a* pathway genes. Salinity stress significantly hampers rice growth and productivity. Transcriptomic analysis of *elh10* and ZH8015 seedlings exposed to salt stress for 24 h identified 5150 differentially expressed genes (DEGs) at the seedling stage, predominantly linked to stress response pathways. *Ehd2* was revealed as a modulator of salt tolerance, likely through the regulation of ion transport, enzyme activity, and antioxidant systems. This study establishes *Ehd2* as a pivotal factor in promoting heading while negatively regulating salt tolerance in rice.

## 1. Introduction

Rice is a vital cereal crop that is crucial to addressing global food security challenges. Heading date is a key agronomic trait, with precocious and excessively delayed heading adversely impacting yield. A proper heading date ensures regional and seasonal adaptability, optimizing reproduction and yield potential [1]. The heading date is coordinated by endogenous external environmental cues [2]. Endogenous factors are strictly controlled by the biological clock, while external signals include photoperiod and temperature. The former has a greater influence on rice heading date [3,4].

Two main photoperiodic pathways govern the transition from vegetative to reproductive growth in rice: the first is the *OsGI*-*Hd1*-*Hd3a*/*RFT1* (*GIGANTEA-Heading date 1*-*Heading date 3a*/*RICE FLOWERING LOCUS T 1*) pathway, orthologous to the *GI*-*CO*-*FT* (*GIGATEA*-*CONSTANS*-*FLOWERING LOCUS T*) pathway in *Arabidopsis* [5]. The other is the unique *Ehd1* (*Early heading date1*)-centered pathway in rice [6]. Both pathways integrate to regulate downstream florigen genes. *Ehd1* promotes heading by inducing *Hd3a* and *RFT1* under both long-day (LD) conditions and short-day (SD) conditions.

Numerous genes modulate *Ehd1* within a complex regulatory network. Core genes include *Hd1*, *Ghd7* (*Grain number, plant height, and heading date 7*), *DTH8* (*Days to heading on chromosome 8*), and *OsPRR37* (*PSEUDO-RESPONSE REGULATOR 37*) [7]. *Hd1* promotes heading under SDs but delays it under LDs [8]. However, in the background of *ghd7* or *dth8*, *Hd1* induces heading regardless of LD and SD [9]. Ghd7 and DTH8 interact with Hd1 to form a repressive complex, delaying heading in LDs by repressing *Ehd1* expression [10]. Similarly to *Ghd7*, *DTH8* can convert the flowering promotion functions of *Hd1* to repress flowering under LD conditions [11,12]. *OsPRR37* is essential for interfering with the function of *Hd1* and cooperates with *DTH8* and *Hd1* to suppress LD heading [13]. Furthermore, Ghd7 and OsPRR37 undergo phosphorylation by Hd16, enhancing *Hd1*’s floral inhibition function under LD [14]. Moreover, Hd17 (also known as OsELF3-1) works with OsELF4s and OsLUX to form a transcriptional repression complex, reducing transcription of *Hd1* and *Ghd7* [15]. Two basic helix-loop-helix (bHLH) transcription factors, HBP1 (Hd1 Binding Protein 1) and POH1 (Partner of HBP1), form homo- or heterodimers to directly bind the promoter of *Hd1* to promote its expression [16].

Recent studies have uncovered novel molecular mechanisms regulating the *Ehd1*-*Hd3a* pathways in rice. The WD40 domain-containing protein EHD5 (Early heading date 5) interacts with Roc4 (Rice outermost cell-specific gene 4) and DTH8, disrupting the inhibitory function of the Ghd7-Ghd8 complex. This interference releases the suppression of *Ehd1*, thereby promoting flowering [17]. Similarly, EHD6 (Early heading date 6), an RNA-binding protein, recruits the m6A reader YTH07 into ribonucleoprotein (RNP) granules via phase separation. This process sequesters *OsCOL4* (*CONSTANS-like 4*) mRNA, reducing the abundance of this flowering repressor and promoting heading through the *Ehd1*-*Hd3a* pathway [18]. *OsWRKY11* exhibits dual functions in heading regulation. Under normal conditions, OsWRKY11 induces heading by directly activating *OsMADS14* and *OsMADS15* expression. However, when OsWRKY11 protein levels increase, it forms a ternary complex with DTH8 and Hd1, which suppresses *Ehd1* transcription and delays heading [19]. Furthermore, the blue light photoreceptor OsCRY2 (Cryptochrome 2) interacts with the transcription factor OsCIBL1 (CRYPTOCHROME-INTERACTING BASIC HELIX-LOOP-HELIX 1) to activate the *Ehd1*-*Hd3a* flowering pathway by directly binding the *Ehd1* promoter [20]. These findings highlight the intricate crosstalk between *Hd1*-*Hd3a* and *Ehd1*-centered pathways, revealing a sophisticated photoperiodic flowering regulation network that adapts rice to diverse regions.

Soil salinization on irrigated land poses a significant challenge to crop yields [21,22]. Rice, being highly sensitive to salt stress, suffers severe growth and developmental impairments—particularly during the seedling and reproductive stages—due to excessive sodium (Na^+^) accumulation [23]. To combat salt stress, plants employ adaptive mechanisms such as osmotic homeostasis (e.g., accumulation of proline, polyols, and sugars), ionic balance (Na^+^/K^+^ homeostasis), and antioxidative defenses (e.g., CAT, APX, GST, and NADPH activity) [24,25,26].

Numerous transcription factors have been found to regulate rice salt tolerance [27]. For instance, the WRKY family member OsWRKY53 negatively regulates salt tolerance by directly inhibiting *OsMKK10.2* and *OsHKT1;5* (*HIGH-AFFINITY POTASSIUM TRANSPORTER 1;5*) expression [28]. Among the MYB transcription factors, the R2R3-MYB subfamily, comprising 117 genes across 12 chromosomes, includes *OsMYB2-115*, which has been identified as vital for salt stress tolerance through genome-wide analyses [29]. Other MYB family members, such as *MYB3R*, *OsMYB36a/b/c*, OsMYB39a, OsMYB41, *OsMYB92a/b*, and *OsMYBc*, are also implicated in salt stress responses [30,31]. The bZIP transcription factor OsbZIP72 enhances salt tolerance by activating *OsHKT1;1* expression in rice [32], while OsbZIP23 activates *UGT2* by directly binding to its promoter [33]. Additionally, GPX1 (GLUTATHIONE PEROXIDASE1) mediates oxidative modification of bZIP68 to enhance the transcription of *COR413-TM1*, *OsDREB1A*, and *OsDREB1B*, and positively regulate the ABA-independent salt stress response [34].

The plant-specific IDD (Cys-2/His-2-type zinc finger) domain transcription factors are integral to various processes, including flowering time, plant architecture, root and seed development, hormone signaling, and responses to biotic and abiotic stresses [35]. Ehd2 (Early heading date 2)/OsID1 (INDETERMINATE 1)/RID1 (RICE INDETERMINATE 1), a ZmID1 ortholog, along with OsIDD4, binds the “TTTGTC” motif in *Hd3a* or *RFT1* promoters to regulate flowering [36,37,38,39,40]. OsIDD10 activates key genes, including *AMT1;2* (*Ammonium transporter 1;2*), *GDH2* (*Glutamate dehydrogenase 2*), *CIPK9* (*Calcineurin B-like protein-interacting protein kinase 9*), and *CIPK14*. This activation is associated with ammonium absorption and nitrogen metabolism in roots, exerting a crucial function in plant growth and development [41,42]. OsIDD3 contributes to cold stress tolerance by directly binding the *CBF1* promoter [43]. Moreover, the OsIDD3-OsIDD13-OsIDD14/LPA1 complex enhances rice’s defense against sheath blight by regulating *PIN1a* and *PIN1b* expression [44,45,46]. However, the role of IDD domain transcription factors in salt tolerance remains unclear.

In this study, we identified a series of IDD domain transcription factor genes, which are novel allele of *Ehd2*/*OsID1*/*RID1*/*Ghd10*. These mutants exhibited late heading phenotypes under both long-day and short-day conditions but showed enhanced salt tolerance in rice. RNA-seq analysis revealed that differentially expressed genes (DEGs) were primarily enriched in stress response pathways. *Ehd2* was further found to mediate salt responses by regulating ion transport, enzyme activity, and antioxidant pathways. These findings underscore the pivotal negative role of *Ehd2* in salt tolerance, highlighting its importance in stress adaptation mechanisms.

## 2. Results

### 2.1. Rice elh5 Mutant Exhibits an Extremely Late-Heading Phenotype

**Figure 1:**
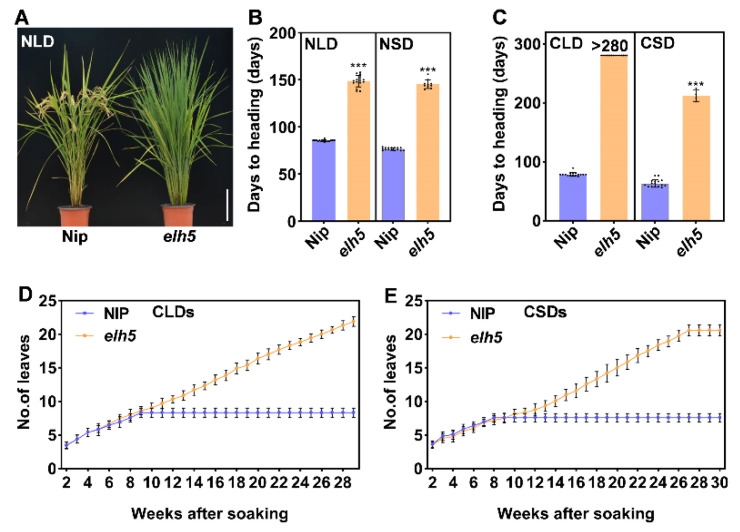
Phenotypic identification and leaf emergence rate statistics in Nip and *elh5*. (**A**) Phenotypic comparison of wild-type (Nip) and mutant (*elh5*) plants grown under natural conditions in Hangzhou, with phenotypic images taken at the mature stage of Nip (scale bar = 20 cm). (**B**,**C**) Heading dates of WT and *elh5* under NLDs and NSDs (**B**), and CLDs and CSDs (**C**). Statistical data are shown as mean ± SD; *** indicates significant differences (*p* < 0.001). (**D**,**E**) Statistics on leaf emergence rate for Nip and *elh5* under CLDs and CSDs, respectively. Data represent mean ± SD (*n* = 15).

To investigate the molecular mechanisms underlying heading date regulation in rice, we identified an extremely late-heading mutant from a Nipponbare (Nip) mutant library generated using ethyl methanesulfonate. The mutant, designated as *elh5* (*extremely late heading 5*), displayed an extended vegetative phase under both natural long-day (NLD) and short-day (NSD) conditions. Compared to Nip, *elh5* mutants delayed heading by approximately 63.1 days under NLDs in Hangzhou and 71.1 days under NSDs in Hainan (Figure 1A,B).

Under controlled photoperiodic conditions, *elh5* mutants exhibited an even more pronounced delay. In growth chambers with controlled long-day (CLD) conditions, Nip plants headed at 78.4 ± 3.2 days, while *elh5* mutants did not head for over 280 days. Under controlled short-day (CSD) conditions, *elh5* mutants delayed heading by 149.0 days, with a heading date of 212.0 ± 8.1 days compared to 63.0 ± 6.2 days for Nip (Figure 1C). This phenotype highlights the indispensable role of the gene disrupted in *elh5* mutants in regulating flowering in rice.

An analysis of leaf emergence rates revealed no significant differences between *elh5* mutants and Nip plants under CLD or CSD conditions before heading (Figure 1D,E). This suggests that the extremely late-heading phenotype of *elh5* results from a prolonged growth phase rather than inhibited growth under either photoperiodic condition.

### 2.2. Map-Based Cloning Identifies the elh5

**Figure 2:**
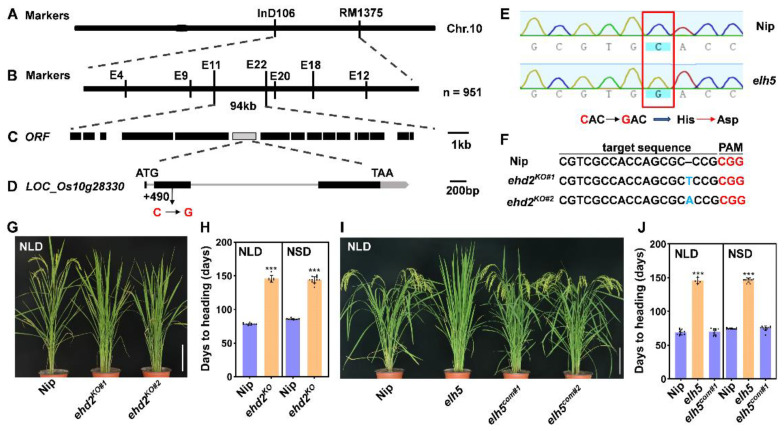
Map-based cloning and functional verification. (**A**–**E**) Genetic linkage analysis localized the target gene to chromosome 10, which was fine-mapped to a 94 kb region containing sixteen putative ORFs. Sequencing revealed a mutation at position 490 in the coding region. (**F**) CRISPR/Cas9-mediated mutagenesis of *Ehd2*, with red letters indicating the PAM sequence and blue letters indicating the insertion. (**G**,**H**) Phenotype and heading dates of WT and two independent *ehd2*^KO^ lines. (**I**,**J**) Phenotype and heading dates of two independent *elh5*^com^ complementation lines. Scale bar = 20 cm; *** indicates significant differences (*p* < 0.001).

To identify the gene responsible for the *elh5* phenotype, we employed map-based cloning. The *elh5* mutant was crossed with the *indica* cultivar *NJ11*, and linkage analysis located the target gene between markers InD106 and RM1375 on chromosome 10 using extremely late-heading plants from the F_2_ population (Figure 2A). Fine mapping with seven InDel markers and 951 extremely late-heading individuals (Figure 2B) refined the locus to a 94-kb region between markers E11 and E22, containing 16 open reading frames (ORFs, Figure 2C).

Sequencing analysis revealed a single nucleotide substitution, a C-to-G transition at position 490 in the coding sequence (CDS) of the second exon of ORF6 (*LOC_Os10g28330*). This mutation corresponds to a novel allele of *Ehd2*/*OsID1*/*RID1*/*Ghd10* (Figure 2D,E). When *elh5* mutants were backcrossed with Nip, all F_1_ progeny exhibited normal heading, and the F_2_ population segregated approximately 3:1 (normal heading:late heading = 845:312; χ^2^ = 2.39, *p* = 0.12), indicating that the abnormal phenotype is controlled by a single recessive locus.

To confirm that the loss of *LOC_Os10g28330* function causes the *elh5* phenotype, we used CRISPR/Cas9 to target the second exon of *LOC_Os10g28330* in Nip. Two homozygous mutants, *ehd2*^ko#1^ and *ehd2*^ko#2^, were generated, each carrying distinct insertions in the targeted sequence (Figure 2F). Phenotypic analysis revealed that these mutants exhibited a late-heading phenotype similar to *elh5* under both NLD and NSD conditions (Figure 2G,H).

Additionally, the transformation of a 7.29-kb genomic fragment, including the 2.31-kb upstream region, the coding region, and a 1.94-kb downstream region of *LOC_Os10g28330* from Nip into *elh5* mutants, fully rescued the late-heading phenotype under both NLD and NSD conditions (Figure 2I,J). These findings confirm that the loss of *LOC_Os10g28330* function is directly responsible for the extremely late-heading phenotype of *elh5*.

### 2.3. Alternative Splicing of Ehd2 Influences Rice Heading

**Figure 3:**
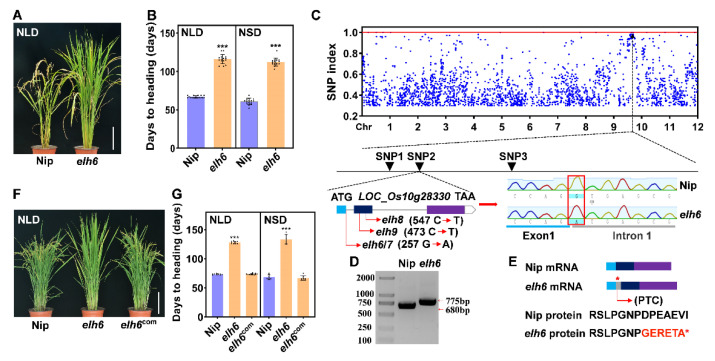
Phenotypic identification and alternative splicing in the *elh6* mutant. (**A**,**B**) Phenotype and heading date of WT and *elh6*. (**C**) Identification of the target gene by MutMap, showing SNP index distributions on chromosomes in *elh6*. Sequencing revealed a mutation at the 5′ splice site of the first intron (red box). (**D**) RT-PCR validation of intron retention in *elh6* between the first and second exons. (**E**) Alternative splicing of mRNA and partial protein sequence, with gray boxes indicating the intron and red asterisks marking premature termination codons, (**F**,**G**) Phenotype and heading dates of WT and two independent *elh6*^com^ complementation lines. Scale bar = 20 cm; *** indicates significant differences (*p* < 0.001).

We identified another mutant allele of *Ehd2*, designated as *elh6*, from the mutant library. Compared to Nip, *elh6* mutants showed delayed heading by 49.2 days under NLD conditions and 51.1 days under NSD conditions (Figure 3A,B). To clone the *elh6* gene, we applied MutMap analysis. Backcrosses of *elh6* with Nip resulted in F_1_ progeny with normal heading. Late-heading F_2_ individuals were used for MutMap analysis, which identified three candidate single-nucleotide polymorphisms (SNPs; SNP1, SNP2, and SNP3) on chromosome 10 with a high SNP index of 1 (Figure 3C and Figure S1). Sequencing revealed a critical SNP (SNP2) at Chr10:14,742,687, a G-to-A transition at the 5′ splice donor site in the first intron of *Ehd2*, following canonical GU-AG splicing rules [47] (Figure 3C). This mutation led to intron retention, introducing a premature termination codon (PTC) within intron 1 (Figure 3D,E).

To verify that the alternative splicing event in *Ehd2* was responsible for the late-heading phenotype, we conducted a genetic complementation assay. Transformation of a 7.29-kb genomic fragment from Nip into *elh6* mutants restored normal heading under both NLD and NSD conditions (Figure 3F,G). This demonstrates that alternative splicing of *Ehd2* disrupts flowering signaling pathways in rice.

### 2.4. Identification of a Series of ehd2 Allelic Mutants

**Figure 4:**
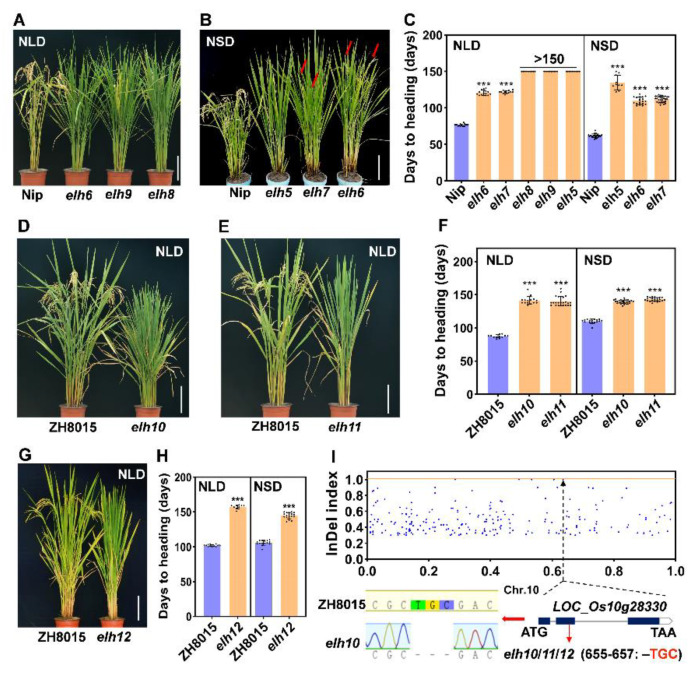
Phenotypic identification and MutMap analysis. (**A**–**H**) Phenotypes and heading dates of *elh5* to *elh9* (**A**–**C**), *elh10* to *elh11* (**D**–**F**), and *elh12* (**G**,**H**) mutants from different genetic backgrounds under NLD and NSD conditions. The red arrow indicates the ears of rice, *** indicates significant differences (*p* < 0.001). (**I**) MutMap analysis identified the target gene by InDel index distributions along chromosomes in *elh12*, with the gene structure and mutation site indicated. Scale bar = 20 cm.

We identified six additional allelic mutants of *Ehd2*, designated as *elh7* to *elh12*. Similarly to *elh6*, the *elh7* mutant exhibited intron retention due to alternative splicing and displayed a late-heading phenotype comparable to *elh6*. The *elh8* and *elh9* mutants contained substitution mutations (C-to-T) at positions 547 and 473, respectively (Figure 3C). Phenotypic analysis under NLDs revealed that *elh8* and *elh9* exhibited late heading similar to *elh5* (Figure 4A–C).

Further, we identified *elh10* and *elh11* mutants from a ZH8015-derived library induced by ethyl methanesulfonate. These mutations resulted in amino acid deletions at positions 655–657 (-TGC, Figure 4I), causing delays in heading by 53.0–54.0 days under NLDs and 29.4–32.9 days under NSDs compared to ZH8015 (Figure 4D–F). Similarly, the *elh12* mutant caused delays of 55.6 days and 38.9 days under NLDs and NSDs, respectively (Figure 4G,H).

To clone the gene responsible for the *elh12* phenotype, we performed map-based cloning and MutMap analyses. A cross between *elh12* and Nip allowed us to map the target locus to markers RM239 and RM25664 on chromosome 10 (Figure S2A). Fine mapping narrowed the region to a 528.3-kb segment between markers M10-10 and M10-8 using 138 late-heading individuals (Figure S2A). MutMap analysis failed to identify SNPs with an index of 1 in the target region (Figure S2B). However, an InDel mutation (Chr10:14,742,191, TGCG-G) with an index of 1 was detected in the second exon of *Ehd2* (Figure 4I). These findings confirm the indispensable role of *Ehd2* in the transition from the vegetative to the reproductive phase in rice.

### 2.5. Ehd2 Encodes an IDD Domain Transcription Factor and Exhibits Transcriptional Activity

Bioinformatic analysis revealed that the rice genome encodes 15 C2H2-type and two C2HC-type zinc finger domain proteins, also known as IDD proteins. Phylogenetic analysis of Ehd2 amino acid sequences showed a close relationship with OsIDD5 (Figure S3A,B). As a zinc finger transcription factor, the transcriptional activity of Ehd2 was investigated using a yeast two-hybrid assay. Segments of the Ehd2 protein (N: 1–104 aa, M: 105–240 aa, C: 241–475 aa) were fused to the yeast GAL4 DNA-binding domain. Yeast growth on defective medium demonstrated that full-length Ehd2 possesses transcriptional activity. However, N- and M-terminal truncated fragments lacked this activity, while the C-terminal fragment exhibited stronger transcriptional activation than the full-length protein (Figure S3C,D).

### 2.6. Expression Pattern of Ehd2 and Subcellular Localization

**Figure 5:**
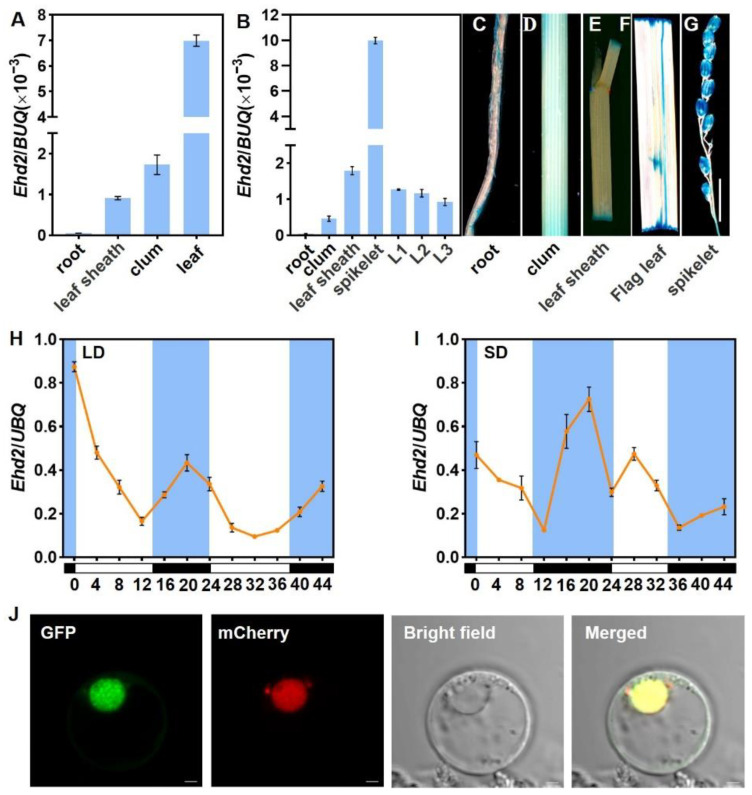
Expression pattern and subcellular localization of Ehd2. (**A**,**B**) mRNA accumulation levels in various tissues at developmental (**A**) and heading stages (**B**). (**C**–**G**) GUS staining of root, culm, leaf sheath, flag leaf, and spikelet. Scale bar = 0.8 cm. (**H**,**I**) Diurnal expression patterns of *Ehd2* under CLDs and CSDs. Blue/black and white boxes indicate dark and light periods, respectively. ZT = 0 marks lights on. Error bars represent standard deviations. (**J**) Subcellular localization of Ehd2. Scale bar = 2 μm.

To investigate the expression pattern of *Ehd2*, we measured its expression in various tissues of Nip plants grown in the field 40 days after sowing and at the heading stage under NLDs. Real-time quantitative RT-PCR (RT-qPCR) analysis showed that *Ehd2* was predominantly expressed in leaves during the vegetative stage, with the highest expression in panicles and the lowest in roots (Figure 5A,B). These results were consistent with β-glucuronidase (GUS) activity assays using *pEhd2::GUS* expression in Nip (Figure 5C–G). This suggests that *Ehd2* regulates leaf flowering during vegetative growth and contributes to panicle development during reproductive growth.

We also examined *Ehd2* expression in leaf blades under various photoperiod conditions. Leaf samples were collected at dawn every 4 hours over one day from plants grown under controlled long-day (CLD) and short-day (CSD) conditions. RT-qPCR analysis revealed rhythmic *Ehd2* expression under both CLD and CSD conditions. Transcript levels decreased during the light period, reaching a minimum at ZT = 12, and increased during the dark period, peaking at ZT = 20 (Figure 5H,I). These findings indicate the importance of *Ehd2* in photoperiodic flowering regulation in rice.

To explore the subcellular localization of Ehd2, we constructed a *d35S::Ehd2:GFP* vector expressing Ehd2 fused to GFP under the control of the cauliflower mosaic virus *d35S* promoter. The vector was co-transformed with a *35S::Ghd7:mCherry* construct, a nuclear marker, into rice protoplasts. The green fluorescence from Ehd2-GFP co-localized with the red fluorescence from Ghd7-mCherry in the nuclei (Figure 5J). This observation, consistent with previous reports, confirms that Ehd2 is a nuclear protein.

### 2.7. elh6 Delayed Heading by Downregulating Floral Pathway Genes

**Figure 6:**
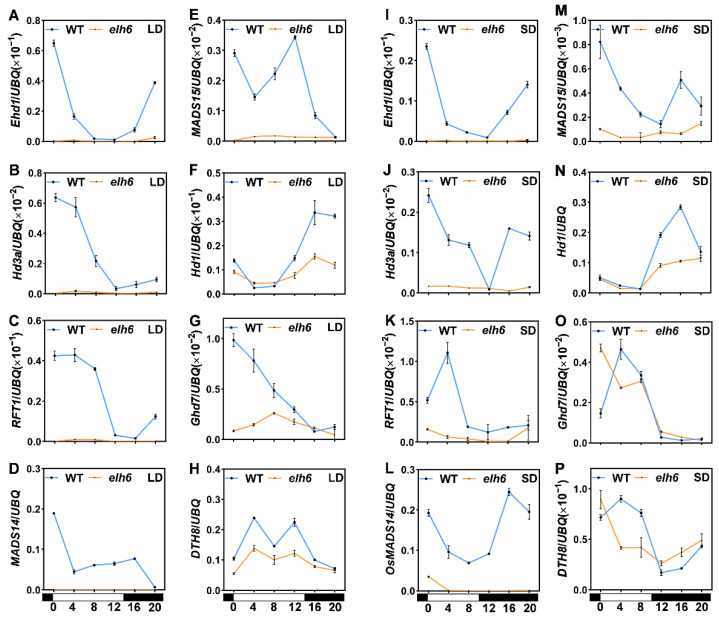
Expression levels of photoperiod flowering genes under CLD and CSD conditions in WT and *elh6*. The black and white boxes indicate dark and light periods, respectively. Samples were collected every 4 h over one day under both LDs and SDs. Relative expression levels of *Ehd1*, *Hd3a*, *RFT1*, *OsMADS14*, *OsMADS15*, *Hd1*, *Ghd7*, and *DTH8* normalized to Ubiquitin are shown under CLDs (**A**–**H**) and CSDs (**I**–**P**). Zeitgeber time (ZT) is indicated, with ZT = 0 marking lights on.

To elucidate the late-heading phenotype of *elh6*, we performed RT-qPCR to analyze the expression levels of flowering time-related genes in both Nip and *elh6* mutant plants under CLD and CSD conditions. The results showed significant downregulation of critical floral pathway genes, including *Ehd1*, *Hd3a*, and *RFT1*, in *elh6* mutants compared to Nip under both photoperiod conditions (Figure 6A–C,I–K). We further assessed the expression of *OsMADS* family genes downstream of the *Ehd1*-*Hd3a* pathway. Consistent with the downregulation of *Ehd1*, *Hd3a*, and *RFT1*, the mRNA levels of *OsMADS14* and *OsMADS15* were also reduced in *elh6* mutants (Figure 6D,E,L,M). These results provide detailed insights into the association between the late-heading phenotype of *elh6* and the downregulation of critical flowering genes.

We also investigated upstream genes involved in the *Ehd1*-*Hd3a* pathway, including *Hd1*, *Ghd7*, and *DTH8*, which are key integrators of rice flowering [9]. The expression levels of *Hd1* were notably lower in *elh6* mutants compared to Nip, particularly during the dark period under both CLD and CSD conditions (Figure 6F,N). Similarly, *Ghd7* and *DTH8* expression levels were reduced in *elh6* mutants, with more pronounced differences observed during the light period than the dark period under both photoperiods (Figure 6G,H,O,P). These findings suggest that *Hd1*, *Ghd7*, and *DTH8* are partially regulated by *Ehd2*, highlighting its crucial role in controlling flowering in rice.

To determine whether *Ehd2* is involved in the regulation of circadian rhythm and photoperception, we analyzed the expression of core clock genes (*OsLUX*, *OsELF3-1*, and *OsCCA1*) and the photoreceptor gene *PHYB* in *elh6* mutants. No significant differences in expression levels were observed between *elh6* mutants and wild-type plants under either CLDs or CSDs (Figure S4). These results indicate that these core clock genes and photoreceptors may not function downstream of *Ehd2* in the regulatory network.

### 2.8. ehd2 Improves Salt Tolerance in Rice Seedlings

IDD domain proteins play important roles in rice growth and development [35]. To explore whether *Ehd2* is involved in salt stress responses, we examined *elh10* and *elh11* mutants with a ZH8015 (*indica*) background, as *indica* rice is more sensitive to salt stress at the seedling stage [48].

**Figure 7:**
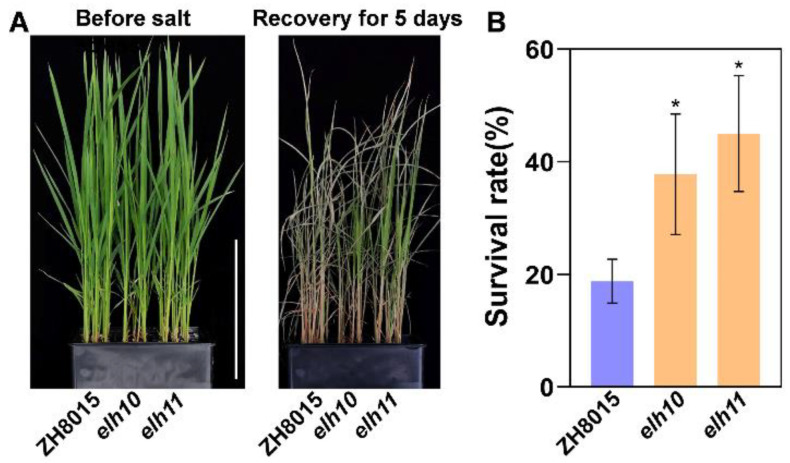
Negative regulation of salt stress by *Ehd2*. (**A**) Phenotypes of ZH8015, *elh10*, and *elh11* before and after salt treatment and recovery. Scale bar = 12 cm. (**B**) Survival rates of ZH8015, *elh10*, and *elh11* after recovery from salt stress. Error bars represent standard deviations; asterisks indicate significant differences (*p* < 0.05).

Both *elh10*/*11* mutants and their wild-type counterpart (ZH8015) were treated with 150 mM NaCl. Before salt stress treatment, no phenotypic differences were observed between the mutants and the wild type. However, after 24 h of salt stress, significant differences emerged. Most ZH8015 seedlings collapsed and exhibited severe wilting, drying, and stunting over five days, while *elh10*/*11* mutants displayed higher survival rates (35.4% and 46.3%, respectively) compared to ZH8015 (17.5%) after five days of recovery under hydroponic conditions (Figure 7A,B). These findings demonstrate that *Ehd2* negatively regulates salt signaling in rice seedlings.

### 2.9. Transcriptomic Analysis of Salt Tolerance in elh10

**Figure 8:**
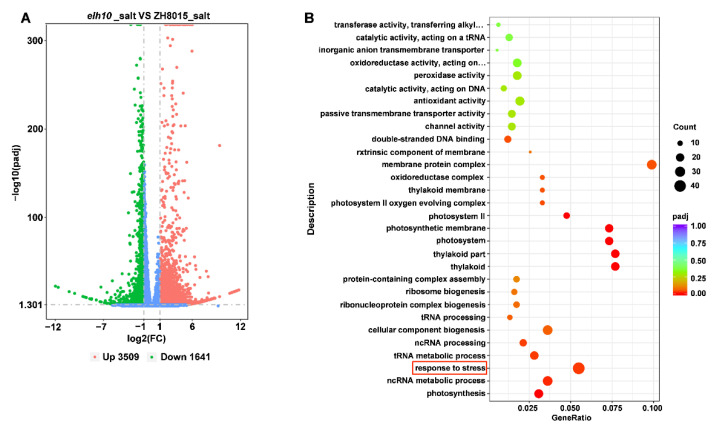
RNA-Seq analysis of *elh10* under salt stress. (**A**) Volcano plot of differential gene expression in *elh10* compared to ZH8015 under salt treatment, the blue dot represents genes with no difference. (**B**) Gene ontology enrichment analysis of upregulated genes was primarily enriched in stress response with red box.

To investigate the regulatory mechanisms underlying *Ehd2*-mediated salt stress responses, we performed RNA-Seq analysis of ZH8015 and *elh10* leaves. A total of 5150 DEGs were identified after 24 h of salt stress, with 3509 upregulated and 1641 downregulated in *elh10* compared to ZH8015 (Figure 8A). Hierarchical clustering revealed global changes in mRNA levels under salt stress in *elh10* mutants compared to ZH8015 (Figure S5).

Gene ontology (GO) enrichment analysis indicated that DEGs were predominantly enriched in stress-response pathways, consistent with *Ehd2*’s role as a negative regulator of salt stress. Compared to ZH8015, *elh10* mutants showed significant enrichments in processes such as “oxidoreductase activity”, “antioxidant activity”, “peroxidase activity”, “passive transmembrane transporter activity”, and “double-stranded DNA binding” (Figure 8B). These findings suggest that *Ehd2* regulates salt stress response via multiple metabolic pathways.

**Figure 9:**
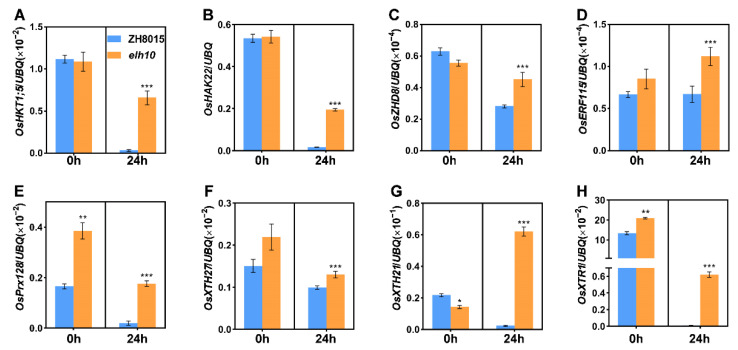
Relative expression of genes in *elh10* and ZH8015 under control and salt stress for 24 h. Relative expression levels of *OsHKT1;5* (**A**), *OsHAK22* (**B**), *OsZHD8* (**C**), *OsERF115* (**D**), *OsPrx128* (**E**), *OsXTH27* (**F**), *OsXTH21* (**G**), and *OsXTR1* (**H**), normalized to *Ubiquitin* under salt stress for 0 h and 24 h. Error bars represent standard deviations; *, **, and *** indicate significant differences (*p* < 0.05, *p* < 0.01, and *p* < 0.001, respectively).

To validate the expression of salt-responsive DEGs, we performed RT-qPCR on eight genes associated with ion transport, enzyme regulation, antioxidant activity, and transcription factors. Notably, under salt stress conditions, genes such as *OsHKT1;5* (sodium ion transporter), *OsHAK22* (potassium ion transporter), *OsZHD8* (zinc finger transcription factor), and *OsERF115* (AP2/EREBP family transcription factor) were significantly upregulated in *elh10* mutants compared to ZH8015 (Figure 9A–D). Additionally, *OsPrx128* (a peroxidase gene) showed higher expression in *elh10* mutants under both control and salt stress conditions (Figure 9E). Genes involved in xyloglucan modification, such as *OsXTH27*, *OsXTH21*, and *OsXTR1*, also exhibited increased expression in *elh10* mutants after 24 h of salt stress (Figure 9F–H). These results align with RNA-Seq data and demonstrate the negative regulatory role of *Ehd2* in these stress-responsive genes in rice.

## 3. Discussion

In this study, we identified a series of late-heading mutants from EMS mutant libraries with diverse genetic backgrounds. The target gene, encoding an IDD domain transcription factor, was found to be allelic with *Ehd2*/*OsID1*/*RID1*/*Ghd10*, which plays a pivotal role in regulating floral transition and yield potential in rice [36,37,38,39,40]. Recent research highlights RID1’s role in regulating *Hd3a* and *RFT1* expression and integrating ABA signaling with photoperiodic pathways [49,50]. Additionally, alternative splicing, particularly intron retention, is a prominent regulatory mechanism in plant growth and stress responses, comprising approximately 83% of alternative splicing events in rice [51].

Research on FLM (FLOWERING LOCUS M) alternative splicing variants has demonstrated their role in regulating the subcellular localization and stability of SVP (SHORT VEGETATIVE PHASE), thereby mediating temperature-responsive flowering in *Arabidopsis* [52]. Similarly, in *Phyllostachys edulis*, the *CONSTANS-LIKE* gene *PeCOL13* was found to suppress flowering through an intron-retention event [53]. However, the role of intron-retained alternative splicing in flowering time regulation in rice remains poorly understood.

In this study, we observed that *elh6* mutant plants exhibited altered pre-mRNA splicing compared to the wild type. Specifically, retention of the first intron in the *elh6* mRNA transcript resulted in a premature termination codon (PTC), leading to a delayed-heading phenotype under both NLD and NSD conditions (Figure 3).

Expression analysis revealed that *elh6* mutants showed nearly undetectable levels of *Ehd1*, *Hd3a*, *RFT1*, *OsMADS14*, and *OsMADS15* under both CLD and CSD conditions (Figure 6A–E,I–M), indicating that *Ehd2* acts upstream of these genes in the rice flowering pathway. Additionally, reduced *Hd1* expression during the dark period and decreased *Ghd7* and *DTH8* expression during the light period in *elh6* mutants under both CLD and CSD conditions (Figure 6F–H,N–P) suggest that these genes are also downstream of *Ehd2*. These findings align with previous studies [36,37,38,39,40].

Earlier research demonstrated that RID1 directly binds to the promoters of *Hd3a* and *RFT1*, but not *Ehd1*, to activate their expression [39]. Since *Ehd1* is repressed by the Ghd7-DTH8-Hd1 complex, *Ehd2* likely influences *Ehd1* via alternate pathways. Moreover, *Ehd2* regulates *Ghd7*, *DTH8*, and *Hd1* through mechanisms beyond photoperiodic flowering. *Ghd7* is known for its pleiotropic effects, influencing not only heading date, plant height, and grain number but also abiotic stress responses such as drought, temperature extremes, and hormone signaling [54,55]. The OsEC-Ghd7-ARE1 module further underscores its role in nitrogen utilization. These findings highlight the complex and multifaceted roles of *Ehd2* and associated genes in rice development.

Previous studies have shown that rice Ehd2/OsID1/RID1/Ghd10 and maize ID1 share a conserved IDD domain and similar tissue-specific expression patterns, and both regulate flowering time [36,37,38,39,40]. Our study also identified multiple *Ehd2*/*OsID1*/*RID1*/*Ghd10* alleles with diverse mutations, resulting in phenotypic differences among mutants (Figure 1A,B, Figure 3A,B and Figure 4A–H), which potentially lead to a phenotypic difference between *elh5* and *elh12* mutants compared to other mutants such as *ehd2*, *osid1*, *rid1*, and *ghd10*. For instance, the *elh5* mutant, with a guanine-to-cytosine substitution at the 490th position in the second exon of the IDD domain, causes an amino acid substitution (Figure 2D,E). Both *elh5* mutants and *ehd2* plants exhibit a similar late-heading phenomenon that is less severe than *rid1* mutants (Figure 1A–C), which never head due to a T-DNA insertion [38].

The *elh6*/*7* mutants, exhibiting PTCs due to intron retention, show weaker late-heading effects than *elh5* (Figure 4A–C). The observed phenotypic differences likely stem from mutation positions within the coding region. In another study, the severity of late-heading phenotypes correlated with reductions in *OsID1* mRNA levels [36]. The *ghd10* mutants have a point mutation in the IDD motif, resulting in a yield-related phenotype characterized by increased panicle length, primary branch number, and number of spikelets per panicle [40]. The phenotypic difference observed may result from mutation differences in the position of the coding region or genetic background.

Recently, advances in CRISPR/Cas9 technology have offered potential solutions for fine-tuning heading dates. Editing cis-regulatory elements in the promoter regions of *Ehd1* can create various weak *Ehd1* alleles with delayed heading and improved yield-related traits [56]. Similarly, high-efficiency multiple promoter-targeting (HMP) gene editing strategies based on CRISPR/Cas9 have been employed to edit the promoter regions of key heading-date genes like *Hd1*, *Ghd7*, and *DTH8*. This editing regulates heading date and expands elite cultivars to higher latitude regions [57]. Given the adverse effects of extreme late heading in *Ehd2* mutants, targeted editing of the *Ehd2* promoter could create variants with reduced expression, enabling the development of elite germplasms with slightly delayed heading and higher yields.

Flowering time is crucial for maximizing yield potential, especially under environmental stress. Plants balance growth with stress tolerance through endogenous molecular signals [58]. While existing models like *OsEC1*-*OsGI* and *STH1*-*Hd1*-*Hd3a* in rice [59,60] and *ZmELF6*-*ZmPRR37* in maize [61] explore the interplay between heading date and salt stress, the underlying molecular networks remain largely unknown. Intriguingly, our study revealed that *Ehd2* regulates flowering and negatively affects salt tolerance by upregulating ion transport-related genes (Figure 6 and Figure 7A,B). For instance, sodium ion transporter *OsHKT1;5*, a key regulator of salt stress tolerance (Figure 9A) [62], and potassium transporter *OsHAK22*, involved in *Ehd2*-mediated salt tolerance (Figure 9B), accumulated in *elh10* mutants, protecting seedlings from Na^+^ overaccumulation and salt stress.

RNA-Seq analysis revealed a significant upregulation of genes associated with enzyme regulator activity, antioxidant functions, and transcription factors, including *OsZHD8*, *OsERF115*, *OsPrx128*, *OsXTH27*, *OsXTH21*, and *OsXTR1* in *elh10* mutants (Figure 9C–H). Previous studies have demonstrated that RID1 binds to the core motif sequence “TTTGTC” in the promoter regions of its downstream target genes [39]. In our analysis, we identified potential RID1-binding elements in the promoter regions of *OsHKT1;5*, *OsHAK22*, *OsXTH21*, and *OsZHD8*, suggesting that these salt tolerance-related genes may be directly regulated by *Ehd2*. However, this hypothesis requires further experimental validation. RID1 is known to regulate flowering by directly binding to the *Hd3a* promoter region and activating its expression [39]. Notably, *Hd3a* loss-of-function mutants exhibited enhanced salt stress tolerance compared to wild-type plants. This was accompanied by delayed flowering under both LD and SD conditions, resulting in an extended vegetative growth phase. This delay provides plants with a “lag effect”, allowing them to better cope with adverse environmental conditions [60]. Given the parallels between *Ehd2* and *Hd3a* in regulating flowering and salt tolerance, we speculate that *Ehd2* also plays a critical role in salinity adaptation. The reduced expression of *Hd3a* in *ehd2* mutants may contribute to decreased salinity sensitivity.

Considering the multifaceted roles of *Ehd2*, a comprehensive understanding of its regulatory network linking salt tolerance and heading date is essential for future research. Increasing the appropriate late heading of rice can enhance its yield by promoting biomass production. Although the loss of *Ehd2* function can enhance rice’s salt tolerance, it often leads to an extremely late heading that ultimately results in reduced yield. Given the negative impact of *ehd2* on rice yield due to extremely late heading, the challenge is how to balance salt tolerance with a slightly delayed heading to ensure optimal yield. Utilizing the CRISPRR/Cas9 system to accurately edit the promoter region of *Ehd2* offers an effective means to address this challenge, potentially generating superior germplasm with both slightly late heading and salt tolerance. In summary, our findings offer valuable insights into the potential molecular mechanisms underlying the interplay between heading date and salt stress, paving the way for further exploration of this intricate relationship.

## 4. Materials and Methods

### 4.1. Plants and Growth Conditions

The *elh5* to *elh12* mutants were induced by EMS mutagenesis in *japonica* cv Nip and *indica* cv ZH8015. These mutants, along with their respective wild-type (WT) plants, were grown under NLD conditions in Hangzhou, China, and NSD conditions in Hainan, China. Additionally, Nip and *elh6* mutants were cultivated in growth chambers under controlled long-day (CLD; 14 h light, 30 °C/10 h dark, 25 °C) and short-day (CSD; 10 h light, 30 °C/14 h dark, 25 °C) conditions, with a light intensity of 300 μmol m^−2^ s^−1^ and 70% relative humidity, to examine heading date and leaf emergence rate.

### 4.2. Cloning of Genes Encoded by elh6 and elh12

We employed a Mutmap and map-based cloning approach, as previously described [15], to identify the target genes. The *elh6* and *elh12* mutants were backcrossed with WT plants to generate an F_2_ segregation population. From this population, 40 F_2_ plants exhibiting extremely late heading were selected for DNA extraction. DNA from these plants was pooled and used to sequence the whole genome, allowing for SNP index calculation at each SNP site. Additionally, *elh6* and *elh12* mutants were crossed with ZH8015 and Nip for linkage analysis, respectively. The combination of high SNP index values and linkage analysis enabled the identification of the target gene.

### 4.3. Vector Construction and Plant Transformation

A 7.29-kb genomic DNA fragment from Nip, comprising a 2.31-kb upstream region, the coding region, and a 1.94-kb downstream region, was cloned into the pCAMBIA1300 vector at the *EcoR* I site. The CRISPR/Cas9 knockout system was employed to generate *ehd2*^ko^ mutants, following the protocol described previously [63]. The 2.31-kb upstream fragment of *Ehd2* was amplified from Nip genomic DNA and inserted into the pCAMBIA1305 vector at the *EcoR* I and *Nco* I sites to create a GUS reporter gene construct. This construct was then transformed into the Nip background. The full-length *Ehd2* CDS was fused with the N-terminal of the pAN580 vector at the *Xba* I site, while the *Ghd7* CDS was fused with *mCherry* at the *EcoR* I site, generating recombinant plasmids for the nuclear marker. The expression vector was transformed into rice protoplasts for fluorescence signal observation. The primers used for plasmid construction are listed in Table S1. Transgenic plants were produced via *Agrobacterium*-mediated transformation of rice calli.

### 4.4. Rice Protoplast Transformation

Rice protoplasts were isolated from 10-day-old Nip seedlings and transfected using the PEG-mediated method as previously described [64]. After co-transformation of *Ehd2*-*GFP* and *Ghd7*-*mCherry* constructs into protoplasts, the protoplasts were incubated in darkness for 16–24 h before the fluorescent signals were examined using a confocal microscope.

### 4.5. RNA Extraction and Real-Time Quantitative RT-PCR (RT-qPCR)

Secondary leaves from Nip and *elh6* mutants grown under CLD (50 days) and CSD (40 days) conditions were collected for total RNA extraction using a FastPure Universal Plant Total RNA Isolation Kit (Vazyme, Nanjing, China) following the manufacturer’s instructions. RNA was reverse-transcribed using Hifair^®^ V One-step RT-gDNA Digestion SuperMix for qPCR (Yeasen, Shanghai, China). RT-qPCR was performed with a SYBR Premix Ex Taq Kit (Takara, Shiga, Japan), following the manufacturer’s guidelines. The relative mRNA levels of the target genes were normalized to *Ubiquitin* using the 2^–△△CT^ method, with two biological replicates and three technical replicates.

### 4.6. Autoactivation Activity Assay

To investigate the autoactivation activity of Ehd2, the full-length *Ehd2* CDS was amplified from Nip cDNA, and five truncated fragments (Ehd2-N: 1–104, Ehd2-M: 105–240, Ehd2-C: 241–475, Ehd2-∆C: 1–240, and Ehd2-∆N: 105–475) were cloned into the pGBKT7 vector at the *EcoR* I site (Clontech, Takara). The BD constructs were transformed into Y2HGold yeast strains, which were grown on selective medium (SD-Trp) for 4 days at 28 °C. Positive yeast colonies were then spotted on selective media (SD-Trp/-His/-Ade and SD-Trp/-His/-Ade/AbA/X-α-gal) and incubated for 5 days at 28 °C to observe growth and coloration.

### 4.7. GUS Staining

*pEhd2::GUS* transgenic plants were grown under NLD conditions. Various tissues (root, culm, leaf, leaf sheath, and spikelet) were collected at the reproductive growth stage. The tissues were subjected to vacuum treatment to allow the GUS staining solution to infiltrate, followed by incubation at 37 °C in the dark until staining ceased. The tissues were decolorized in 92% alcohol, and this process was repeated three times. Chlorophyll was removed by boiling the tissues in 92% alcohol, then cooling them to room temperature. The blue-stained tissues were photographed or stored in 70% alcohol for future use.

### 4.8. Salt Stress Treatment

For salt stress treatment, seeds of ZH8015, *elh10*, and *elh11* were first dried at 50 °C for 2 days. The seeds were then surface-sterilized with 75% ethanol for 5 min, washed three times with sterile water, and treated with sodium hypochlorite solution containing an effective chlorine concentration of 2.84% (Sangon Biotech Co., Ltd., Shanghai, China; Lot number J201WA7001) for 30 min, followed by three washes with sterile water to break dormancy. The seeds were placed on soaked filter paper at 37 °C for several days until they germinated. Germinated seedlings were transferred to a 96-well hydroponic box containing Yoshida’s solution and grown in a growth chamber under CLD conditions for 3 weeks, with nutrient solution changes every 5 days. After 3 weeks, the seedlings were treated with 150 mM NaCl solution for 5 days. Phenotypic observations were made after 5 days of recovery, with three biological replicates.

### 4.9. RNA-Seq Analysis

RNA-seq was performed as described previously [65]. Total RNA samples were extracted from 3-week-old ZH8015 and *elh10* seedlings (24 h after 150 mM NaCl treatment) using a FastPure Universal Plant Total RNA Isolation Kit (Vazyme, Nanjing, China). Purified total RNA was used to construct libraries, which were sequenced using the TruSeq PE Cluster Kit v3-cBot-HS (Illumina, San Diego, CA, USA). Sequencing was performed by Novogene Bioinformatics Technology (Beijing, China). Raw reads were filtered and aligned to the rice reference genome using HISAT2, providing the location information for the clean reads.

## 5. Conclusions

In this study, a series of late-heading mutants, ranging from *elh5* to *elh12*, were isolated using a combination of MutMap and map-based cloning methods. The candidate gene identified was a novel allele of *Ehd2*/*OsID1*/*RID1*/*Ghd10*. CRISPR/Cas9 knockout and complementation assays confirmed its role in regulating heading date. The *elh6* mutant represents a novel allelic variant that results in intron retention due to alternative splicing. Complementation assays confirmed the role of alternative splicing in *Ehd2*’s heading regulation function. RT-qPCR analysis revealed that *elh6* delayed heading by downregulating the *Ehd1*-*Hd3a* floral pathway genes. *Ehd2* encodes an IDD domain (Cys-2/His-2-type zinc finger) transcription factor that plays key roles in the response to abiotic and biotic stresses. When subjected to salt stress, *Ehd2* demonstrates negative regulation on rice salt tolerance. Transcriptomic analysis conducted on *elh10* and ZH8015 seedlings under 24 h of salt stress identified 5150 DEGs. Gene ontology analysis revealed that these DEGs were mainly associated with stress response pathways, strongly suggesting that *Ehd2* is a key regulator of the salt stress response in rice. Our findings not only offer insight into the potential molecular mechanisms linking heading date and salt stress but also pave the way for future rice breeding programs aimed at enhancing both heading adaptability and salt tolerance, presenting a significant opportunity for further exploration.

## Data Availability

The data used in this study are available with in the article and its accompanying Supplementary Materials. Additionally, the RNA-Seq data used in this study have been deposited in the National Center for Biotechnology Information (NCBI) data repository with the accession number PRJNA1212471.

## Supplementary Materials

The following supporting information can be downloaded at: https://www.mdpi.com/article/10.3390/plants14020297/s1, Figure S1: Distribution of single-nucleotide polymorphisms (SNP) index on chromosome 10. Figure S2: Identification of target genes in elh12 mutant via map-based cloning and MutMap. Figure S3: Phylogenetic analysis and transcriptional activity of Ehd2. Figure S4: Relative expression levels of rhythmic clock genes under LDs and SDs in WT and elh6. Figure S5: Heatmap of differentially expressed genes in elh6 compared to ZH8015. Table S1: Primers used in this study. The accession numbers are as follows: *Ehd1* (LOC_Os10g32600), *Hd1* (LOC_Os06g16370), *Ghd7* (LOC_Os07g15770), *DTH8* (LOC_Os08g07740), *Hd3a* (LOC_Os06g06320), *RFT1* (LOC_Os06g06300), *OsMADS14* (LOC_Os03g54160), *OsMADS15* (LOC_Os07g01820), *OsLUX* (LOC_Os01g74020), *OsELF3-1* (LOC_Os06g05060), *Phyb* (LOC_Os03g19590), *OsCCA1* (LOC_Os08g06110), *OsHKT1;5* (LOC_Os01g20160), *OsHAK22* (LOC_Os07g01214), *OsXTH27* (LOC_Os10g02770), *OsXTH21* (LOC_Os07g29750), *OsXTR1* (LOC_Os11g33270), *OsPrx128* (LOC_Os10g39170), *OsERF115* (LOC_Os08g41030), *OsZHD8* (LOC_Os04g35500).
