# Supplementary material for: A Series of Novel Alleles of Ehd2 Modulating Heading and Salt Tolerance in Rice
Source: Plants (Basel). 2025 Jan 20;14(2):297. doi: 10.3390/plants14020297 (PMC11769052; doi:10.3390/plants14020297)
Supplement: Supplementary file 1 [file plants-14-00297-s001.zip › plants-3418500-supplementary.pdf]

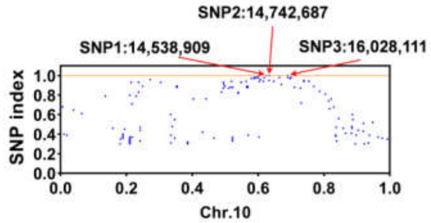

Figure S1. Distribution of single-nucleotide polymorphisms (SNP) index on chromosome 10. The Y-axis represents the SNP index, and the X-axis represents the relative position of SNP loci on chromosome 10. Three SNP sites with an SNP index of 1 are indicated.

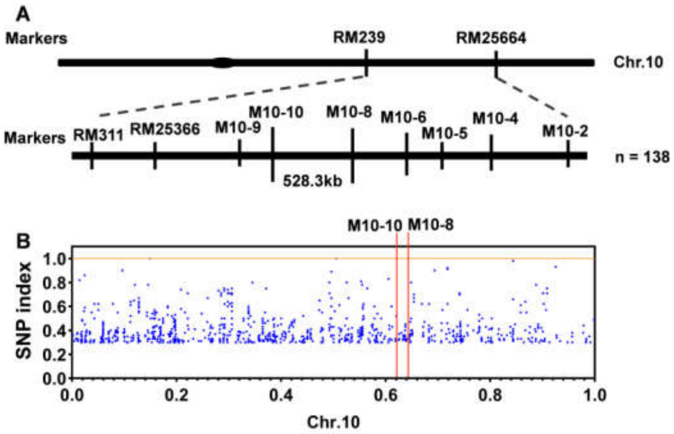

Figure S2. Identification of target genes in *elh12* mutant via map-based cloning and MutMap. (A) Based on linkage analysis, the target gene locus was mapped to a 528.3-kb region on chromosome 10 between M10-10 and M10-8. (B) Distribution of SNP index on chromosome 10 between M10-10 and M10-8.

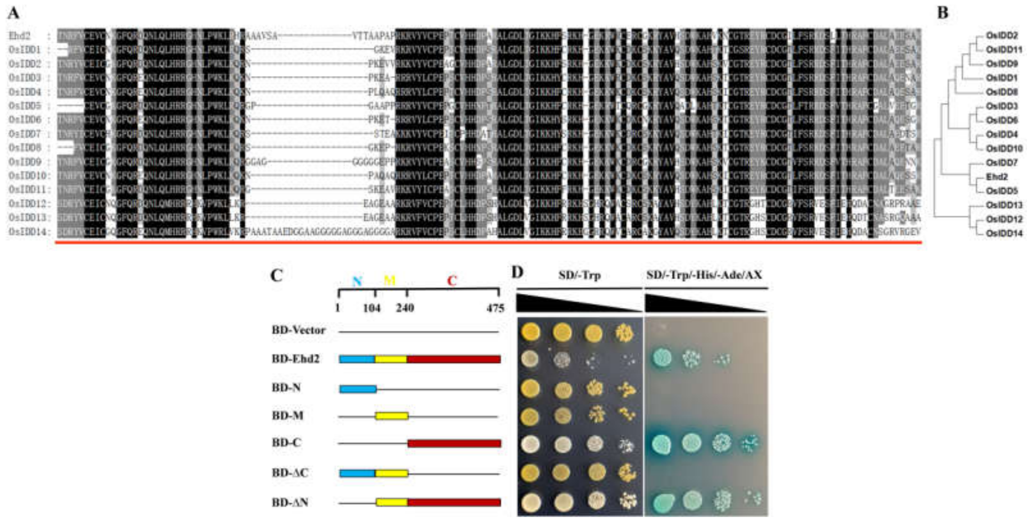

Figure S3. Phylogenetic analysis and transcriptional activity of Ehd2. (A) Multiple sequence alignment of IDD (C2H2/C2H2/C2HC/C2HC) domain homologous proteins using ClustalW and GenDoc. The conserved IDD domain is underlined in red. (B) Phylogenetic tree of Ehd2 constructed

using the neighbor-joining method. (C-D) full-length Ehd2 protein sequence and truncated peptide fragments fused in pGBKT7. Yeast cells with pGBKT7 constructs were incubated on selective media (SD/-Trp) and (SD/-Trp/-His/-Ade/AX), respectively. AX indicates AbA and X-α-gal.

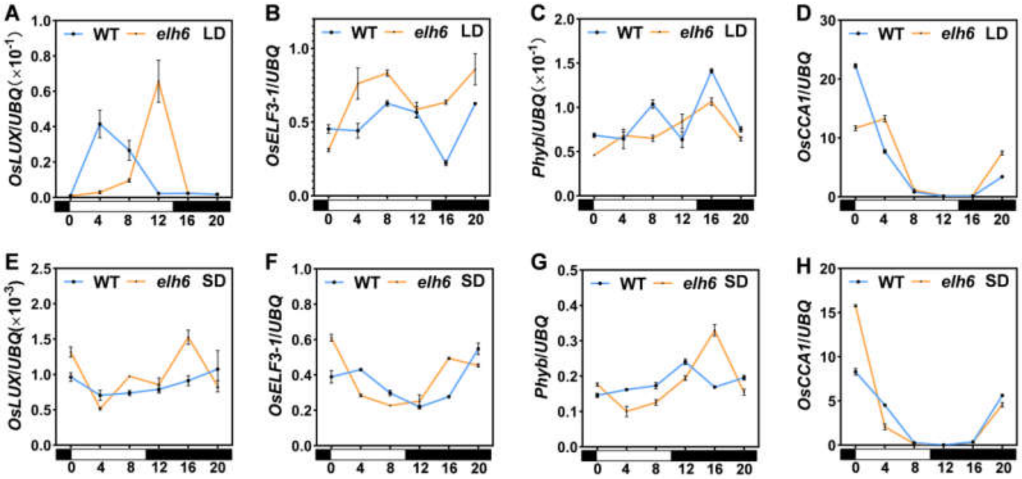

Figure S4. Relative expression levels of rhythmic clock genes under LDs and SDs in WT and *elh6*. The black and white boxes represent dark and light periods, respectively. Expression levels of *OsLUX*, *OsELF3-1*, *Phyb*, and *OsCCA1* normalized to rice *Ubiquitin* are shown under LDs (A-D, I-L) and SDs (E-H). Zeitgeber time (ZT) is indicated, with ZT = 0 marking lights on.

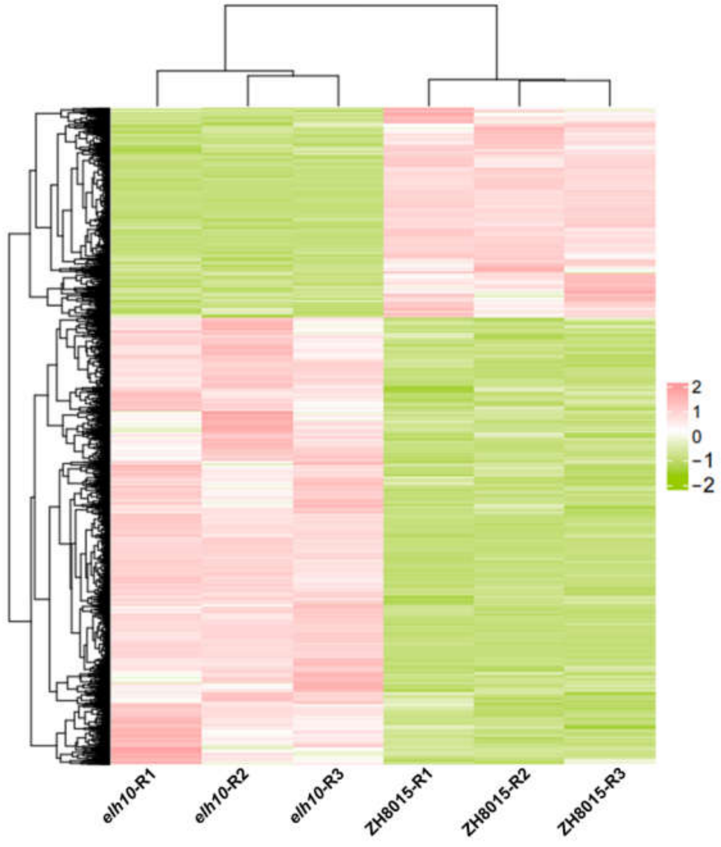

Figure S5. Heatmap of differentially expressed genes in *elh6* compared to ZH8015. The scale bar

indicates fold changes (log_2 value). Three-week-old seedlings were treated with salt stress for 24 hours, and the leaves were collected for RNA-Seq analysis.

Table S1. Primers used in this study.
Primers used for map-based cloning

Primer	5'	3'
InD106	AATGACAAGGCCGACGATAG	TATTACCCAGGCCAACCTGT
RM1375	CTACACGCGCAAACTCTGTC	ATGAAGGTCTAGGCTGCACC
E4	TTGGTTTCCTAGCTTGGCCTACG	TAGAATGCGATGCGAGACAGTGG
E9	TACGTGTCCTTGTGCCTGAAA	TGACAAATGAACTGAGAACGTGAA
E11	CGGAGACAACGAATGCTCAGTGG	CCAGATCTGCGAGGGAGGATGG
E12	TGGGCCAACCTTAGGATCAA	TGCTGTCTATCACATCTATCACCG
E18	TATGAGTGAGTTGAAATAAGAAACT	TCTAATGTCGTAACAACTTACTTTT
E20	TCTGAACCTTTATTGACCCACT	GGGTCTTGAAACTCTAGGATTG
RM239	TACAAAATGCTGGGTACCCC	ACATATGGGACCCACCTGTC
RM25664	CCAGCTGTTAATTAGCATGTGTGAGC	CAGCAAAGGCGTACGTATCTCG
RM311	TGGTAGTATAGGTACTAAACAT	TCCTATACACATACAAACATAC
RM25366	TCGGTCTCTGTGCCGTGATTAGG	CACCAGCGCAGCAACTAACATCC
M10-9	GAGTGGTACGCCGCGTAGAA	CCTCCAAAACCAGCAGATCG
M10-10	CACTACCATCGCAACCACTATTG	GCGTTAGCCATAAACGGATTATTCTC
M10-8	TTCTTTGGCCAGCCCAAACA	CGCTGCTGTTGCAAGAGATG
M10-6	GTCATGTCACTAAGGATTAGGAATGG	GGAATTGGCACAACAGTACAAGAG
M10-5	GCGGCAGAAGGAAATCGATC	GCTCATGAGCTTAAACGAGCTG
M10-4	CCATCTTTTAGCTGAAGTACCTAAAC	CTTTGATCAGGTGTATTTGAGACG
M10-2	GTTTTCAAATGTGACTGACAACG	TCGTCGACATCCTCTCATCC

Primers used for vector construction

Primer	5'	3'
Ehd2-com	ccatgattacgaattcAGAGCCCACAAAGCCAAAGG	taccgagctcgaattcAGAAACGGATACTTGCTTCCT
Ehd2-KO	ggcaGACGAACCGCTTCGTCTGCG	aaacCGCAGACGAAGCGGTTCGTC
Ehd2-pAN580	ccggagctagctctagaATGTTGCTGTCTGATCTCTC	cctcgagacgtctctagaGAAGTTGTGGCTCCACGTCT
Ghd7-mCherry	ccgggctgcaggaattcATGTCGATGGGACCAGCAG	atcgataagcttgatatcTCTGAACCATTGTCCAAGCT
Ehd2-BD	atggaggccgaattccATGTTGCTGTCTGATCTCTC	ggatccccgggaattcGAAGTTGTGGCTCCACGTCT
Ehd2-N-BD	atggaggccgaattccATGTTGCTGTCTGATCTCTC	ggatccccgggaattcCTGGTGGCGACGAACCGC
Ehd2-M	atggaggccgaattccTTCGTCTGCGAGGTCTGC	ggatccccgggaattcGGCATCGCAGAAGGCCCT
Ehd2-C	atggaggccgaattccCTAGCTGAAGAGAGTGCA	ggatccccgggaattcGAAGTTGTGGCTCCACGTCT
Ehd2-ΔC	atggaggccgaattccATGTTGCTGTCTGATCTCTC	ggatccccgggaattcGGCATCGCAGAAGGCCCT
Ehd2-ΔN	atggaggccgaattccTTCGTCTGCGAGGTCTGC	ggatccccgggaattcGAAGTTGTGGCTCCACGTCT

Primers used for qRT-PCR

Primer	5'	3'
Ubq-qRT	GCTCCGTGGCGGTATCAT	CGGCAGTTGACAGCCCTAG
Ehd1-qRT	CCTACAGTGATTATGGCTTCA	GTGCTGCCAAATGTTGCTC

Hd1-qRT	GGCGTCAGTGCTTACACAGATT	TCCAGCAGGTGTCAGGATTCT
Ghd7-qRT	GCTTGAACCCAAACACGG	CTCATCTCGGCATAGGCTT
DTH8-qRT	CAGGAGTGCGTGTCGGAGTT	GGTCGTCGCCGTTGATGGT
Hd3a-qRT	GCTCACTATCATCATCCAGCATG	CCTTGCTCAGCTATTTAATTGCATAA
RFT1-qRT	TGACCTAGATTCAAAGTCTAATCCTT	TGCCGGCCATGTCAAATTAATAAC
OsMADS14-qRT	GCAATGGGACCAGACACAAC	CTGCTACATCCTCTATCCTTTCG
OsMADS15-qRT	CCCTACCCTACAGGCTACATA	TAGGAAGCACTAGGTACGTGCTGA
OsLUX-qRT	CCTGAAGAAGGGTCTGGGTC	CTCGCCCATAATACTCGGAT
OsELF3-1-qRT	TGTCGCCCCTTCGTCAA	GGTCTTTTCCCCAGCTCATT
Phyb-qRT	CTCATCTTCAAGGAATCTGAGG	CCTGCTAGAACAAGCATTCAC
OsCCA1-qRT	ATCGACATGATTTGCTTTCCAC	GTCTGAAGTCCTATGGTAGCTC
OsHKT1;5-qRT	CCTGCCACCTTACACCACTT	GCTGTAGTTGATGGGGTCGT
OsHAK22-qRT	CTGACGTCGGTGCTCTACAA	GAGACACAAGCTCCCTCACC
OsXTH27-qRT	CCATCGTTACGCCATTCTTT	GAAAACTCTGCGACGAAAGG
OsXTH21-qRT	ACCATCTGGCTCGACAAAAC	ACGTTGGTCTGGAGGGTGTA
OsXTR1-qRT	GGCGTTCGAGAAGAACTACG	GACAGGTAGAAGGCGGTGAC
OsPrx128-qRT	CGATGCTGTCGTGGTACTTG	AAGCAGTCGTGGAAGTGGAG
OsERF115-qRT	GAGAGGTACGCCCACGAC	TGTCAGGCTCGTTGCAGTAG
OsZHD8-qRT	ATGCACAACCACAAGAACCA	TAGACGCTGATCGGTGAAGG
